# Mastering polymer chemistry in two dimensions

**DOI:** 10.1038/s42004-020-0258-5

**Published:** 2020-01-24

**Authors:** A. Dieter Schlüter

**Affiliations:** Department of Materials, ETH Zürich, Zurich, Switzerland

**Keywords:** Polymers, Two-dimensional materials, Polymer synthesis

## Abstract

Organic 2D materials display valuable properties that are unique from their bulk counterparts, but creating covalent sheets with long-ranging order remains a formidable challenge. Now, reacting complementary monomers right below a surfactant monolayer on water proves to be a powerful method to create organic 2D materials with long-range order.

Creating long-ranging order is arguably one of the greatest challenges in the chemistry of organic 2D materials^1^. A simple back-of-the-envelope calculation considering monomer size and monomer packing shows that already for a 1-μm^2^-sized monolayer sheet the number of bonds that have to be formed between all the monomers is easily on the order of 0.5 million. For sheets in the range of mm^2^ or of wafer size, this number approaches infinity. Synthesizing organic 2D materials is therefore highly challenging, but of importance as they will greatly differ from the known 3D materials, even for similar compositions. This is due to the absence of a bulk phase, the simultaneous presence of two surfaces (on both sheet sides) and the existence of a 1D circumference.

Mastering such complex molecular systems means no less than finding a growth process that connects the monomer length scale with the sheet length scale by bridging several orders of magnitude, ideally with full structural control. Doing so without departure into the third dimension is a formidable challenge. Nature has learned to do this over billions of years. The many inorganic layered 2D materials such as graphite or some silicates impressively testify to this. However, doing the same in a chemistry laboratory under synthetic, i.e. mild, conditions? Almost unheard of. Yes, forming a few bonds in organic molecules or even 10,000 bonds in linear polymers has been developed to very high levels of perfection^2^, but doing the same with millions and billions of bonds within a plane was virtually dreamland.

Recently, an international group of researchers led by Xinliang Feng at Technical University of Dresden, Germany, and Zhikun Zheng, Sun Yat-sen University, China, reported a completely new approach^3^. Aided by a surfactant monolayer at an air/water interface, two pairs of complementary monomer molecules covalently connect to form approximately 2-nm-thick layered 2D polyimide (**2DPI**) and polyamide (**2DPA**) with long-range order (Fig. 1) as proven by aberration corrected selected area electron diffraction (*ac*-SAED). High-resolution transmission electron microscopy imaging (HRTEM) afforded the average crystal domain size range, from a few tenths of a μm^2^ up to a few μm^2^. A layered structure is suggested by step-edges in the 2D material, the height of which corresponds to monolayers.

**Fig. 1:**
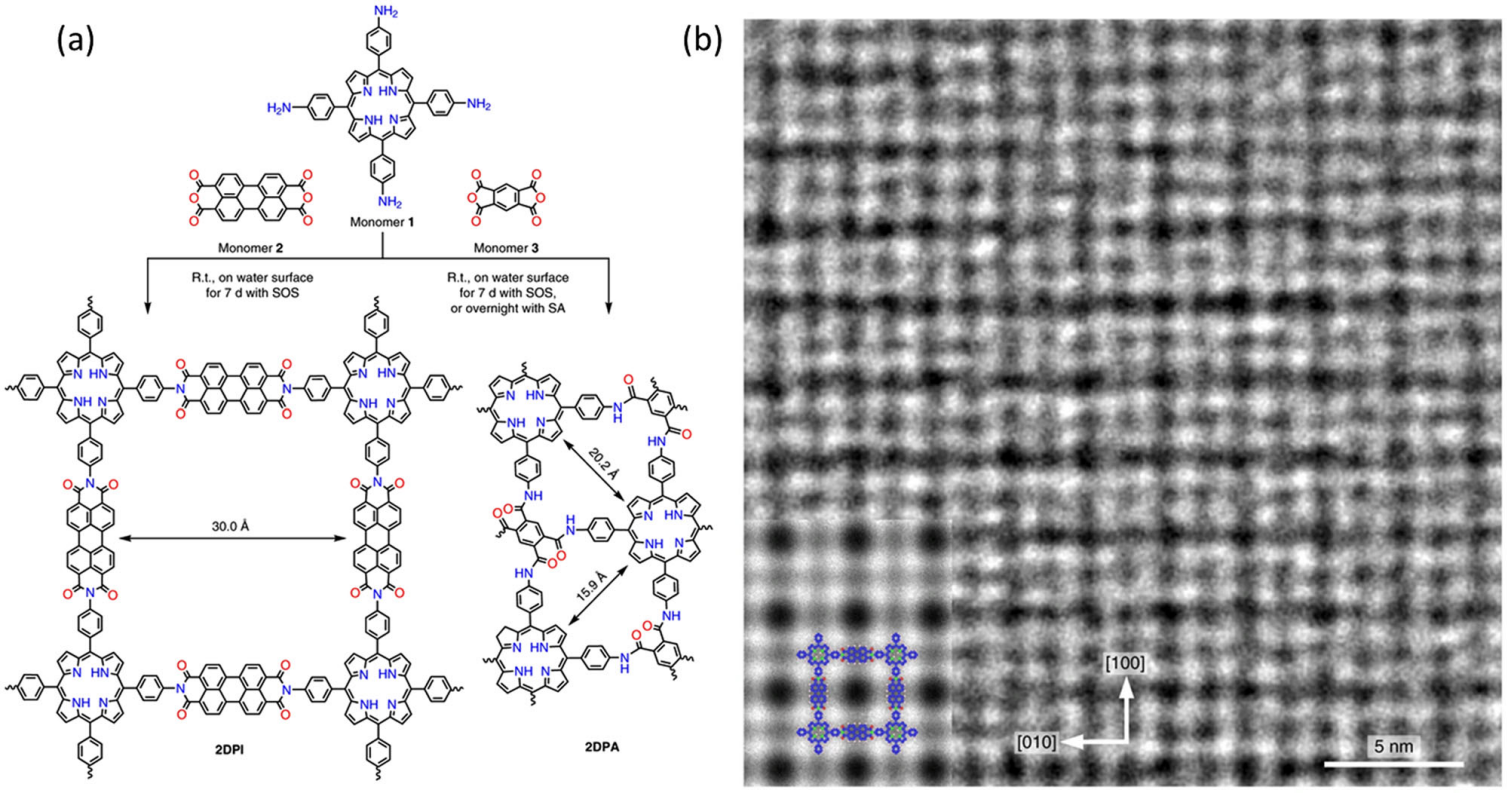
Mastering polymer chemistry in 2D. **a** Synthetic scheme for 2D polymers **2DPI** and **2DPA** from monomers **1** and **2** and monomers **1** and **3**, respectively. **b** AC-HRTEM image of **2DPI**. Inset: a simulated image of **2DPI** along the [001] projection with the structure model overlaid (blue, red, green and white dots represent carbon, oxygen, nitrogen and hydrogen atoms, respectively). Reprinted by permission from SpringerNature^3^.

Putting this remarkable finding into perspective is not easy, primarily because there are no suitable few-layer synthetic organic systems for direct comparison. We can however look to two of the few recently published 2D organic monolayers for comparative discussion^4,5^. Yu Zhong, Jiwoong Park and collaborators from the University of Chicago, USA, reported on the creation of a covalent network, **2DPII**, by the condensation of a tetra-functional porphyrin derivative and a terephthalaldehyde at a pentane/water interface^4^. While the resulting monolayer product was covalently bonded and even wafer-scale in size, in-plane grazing incidence wide angle X-ray diffraction (GIWAXS) measurements on a stack of **2DPII** layers revealed crystalline areas in the range of only about 20 nm (Fig. 2a). Interestingly, the authors compared this scattering result with local structural information based on scanning tunnelling microscopy (STM), which allows for correlation of the experimentally obtained contrast with the molecular structure. The beautifully resolved image in Fig. 2b shows crystalline patches—no mean feat given the chemistry involved—but they are parts of a largely irregular network. Thus, there is still a way to go to achieve long-range order by this synthetic strategy.

**Fig. 2:**
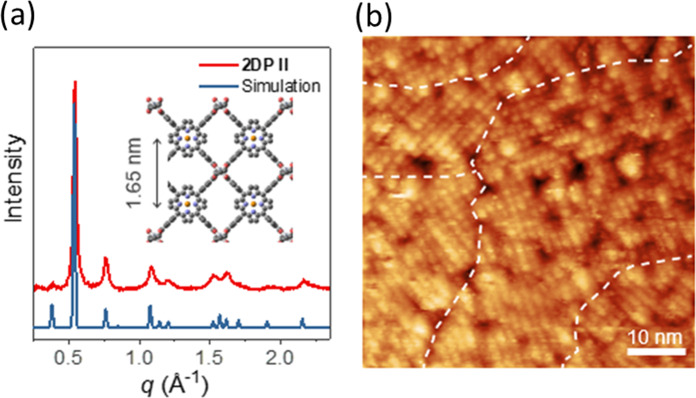
Determining crystallinity in covalent sheets. **a** Experimental and calculated in-plane XRD profiles of **2DPII**. The experiment was conducted on a stacked **2DPII** of 147 layers on sapphire. Inset: crystal structure of **2DPII**. **b** Constant-current STM topography image of multiple crystalline domains of monolayer **2DPII**. White dashed lines manually identify boundaries between different domains (from ref. ^4^). Reprinted with permission from AAAS.

Another recent report detailing a monolayer 2D polymer was published by a group of researchers at ETH Zürich and the University of Basel collaborating with the author of this Comment^5^. Photochemical fixation of a monomer monolayer pre-ordered at an air/water interface gave a crystalline network, the long-range order of which was demonstrated by non-contact mode high-resolution atomic force microscopy (*nc* HRAFM) in ultra-high vacuum after transfer onto highly oriented pyrolytic graphite. This investigation revealed that several few 10,000-nm^2^-sized regions on a specimen of a few tens of a mm^2^ were almost fully covered by monolayer.

A direct comparison of the lateral extension of order achieved in these three cases is challenging because quantification is based on different analytical methods. May it be HRTEM, *ac*-SAED, GIWAXS, STM, or *nc* HRAFM, each technique probes something different and has specific inherent limitations. Thus, each method provides an important piece of structural information, but on its own does not provide a comprehensive and fully representative structural picture, which would form the basis for a proper comparison. Nevertheless, the extension of order achieved in Dresden is clearly larger than that in Chicago and may even surpass the one in Zürich. It marks thus a big step forward in mastering complex bond formation processes. Additionally, the monomer components used for this surfactant-assisted synthesis, the tetraaminoporphyrin derivative **1** and the dianhydrides **2** (for **2DPI**) and **3** (for **2DPA**), are commercially available. This removes the need of having to go through even simple steps of monomer synthesis, which are required for the Zürich 2D polymer^5^. Given the fact that chemical synthesis is often an unsurmountable obstacle for physicists and engineers, this is a plus. Other laboratories are more likely to copy this fascinating work, furthering the exploration of these novel materials towards properties and applications.

Having worked for years on 2D polymers^6^, a subset of organic 2D materials, we have learned how demanding it is to prove chemical structure and long-ranged order (crystallinity) in layered materials. Typically, the quantities are tiny and the layers are sensitive towards tearing and decomposition during analysis, for example when exposed to an electron beam while trying to perform HRTEM imaging or SAED, even at the lowest possible electron dose. Furthermore, given the softness of extremely thin layers of large lateral extension, preparing samples suitable for HRAFM or STM studies can be extremely arduous. It is therefore a particularly admirable feature of the Dresden work that in collaboration with the leading transmission electron microscopy laboratory of Ute Kaiser at the University of Ulm, Germany, Haoyuan Qi was able to obtain impressively resolved *ac* HRTEM images supporting the proposed crystallinity (Fig. 1b). In addition, they could resolve grain boundaries, which enabled estimation of the mentioned domain sizes. In light of the propensity of organic materials to suffer electron beam damage, it is advantageous that the synthesis strategy did not provide monolayers but rather a layered material with a thickness of approximately 2 nm. Such objects are more stable to the electron beam, which is a prerequisite for the fascinating structural insights obtained.

The high level of order accomplished in the covalent 2D materials **2DPI** and **2DPA** now pushes the gate wide open to a variety of pressing investigations, encompassing mechanistic and structural studies and moving towards application. Indeed, although the authors presented structural models for how monomer **1** assembles right beneath the surfactant monolayer, the structural rearrangements required to bring about the reaction between **1** and **2** and **1** and **3**, respectively, are so severe that gaining a mechanistic understanding of this process appears mandatory. This is even more so the case given that the bond formations take place under unprecedentedly mild conditions and would be irreversible under common flask-type conditions. Irreversible bond formation suppresses the feature from which the synthesis of 2D covalent organic frameworks (2D COF)^7^ profits so much: defect healing, which may not be operative here. Thus, this work creates a fantastic opportunity for meaningful, yet non-trivial research towards gaining insight into, for example, how the system accommodates volume changes during synthesis and how it avoids defect formation. It would also be important to improve on availability in terms of mass and to understand the factors that lead to the formation of a layered product, cause the domain formation, and limit the lateral growth. Furthermore, it would be desirable to explore the suitable monomer range and to know whether or not the layers in the few-nm-thick materials **2DPI** and **2DPA** can be exfoliated into monolayers. This last aspect would not only prove that the material is in fact a stack of independent layers (2D polymers)—HRTEM images are two-dimensional projections and cannot determine this—but it would also provide access to samples of large 2D polymers. Such materials are of greatest interest for both the study of polymer physics and for applications. All of the extremely successful concepts and models of polymer physics were developed for linear polymers, and it needs to be seen inasmuch that they can be applied to 2D polymers as well^8^. Concerning applications, it is noted that both monolayers of **2DPI** and **2DPA** and thin layer stacks thereof are potentially attractive for e.g. separation purposes (gases, water desalination, water purification), primarily owing to their mechanical strength and the ordered array of monodisperse nanopores present within extremely thin materials. The fishing net-type structure of 2D polymers brings up yet another synthesis goal: can one make covalent monolayers directly, using the same synthesis strategy, and equip them with electrical conductivity, as inspired by the recently observed crystalline monolayers of (mostly linear) polyaniline^9^?

The synthetic organic 2D polymers and 2D materials made in Dresden, Chicago and Zürich are beautiful examples for how organic synthesis can conquer unexplored territories. Sheet-like macromolecules, which so far have had a Cinderella-like existence, are currently becoming more of a focus in both polymer chemistry and materials chemistry. With a bit of imagination and a portion of persistence, each of the samples described herein can be used to explore burning questions of fundamental and applied science. The beauty of any such endeavour is that the great aesthetic appeal these novel materials have makes one forget all the pain associated with their creation, structural characterization and implementation into functional devices.
